# Social factors matter in cancer risk and survivorship

**DOI:** 10.1007/s10552-018-1043-y

**Published:** 2018-05-30

**Authors:** Lorraine T. Dean, Sarah Gehlert, Marian L. Neuhouser, April Oh, Krista Zanetti, Melody Goodman, Beti Thompson, Kala Visvanathan, Kathryn H. Schmitz

**Affiliations:** 1 Department of Epidemiology, Johns Hopkins Bloomberg School of Public Health and Department of Oncology, Johns Hopkins School of Medicine, 615 N Wolfe St, E6650, Baltimore, MD 21205 USA; 2 College of Social Work, University of South Carolina, Columbia, SC USA; 3 Cancer Prevention Program, Division of Public Health Sciences, Fred Hutchinson Cancer Research Center, Seattle, WA USA; 4 Behavioral Research Program Division of Cancer Control and Population Sciences, National Cancer Institute, Bethesda, MD United States; 5 Epidemiology and Genomics Research Program, Division of Cancer Control and Population Sciences, National Cancer Institute, Bethesda, MD USA; 6 Department of Biostatistics, College of Global Public Health, New York University, New York, NY USA; 7 Department of Public Health Sciences, College of Medicine, Pennsylvania State University, Hershey, PA USA

**Keywords:** United States, Breast cancer, Social determinants, Race/ethnicity, Disparities

## Abstract

Greater attention to social factors, such as race/ethnicity, socioeconomic position, and others, are needed across the cancer continuum, including breast cancer, given differences in tumor biology and genetic variants have not completely explained the persistent Black/White breast cancer mortality disparity. In this commentary, we use examples in breast cancer risk assessment and survivorship to demonstrate how the failure to appropriately incorporate social factors into the design, recruitment, and analysis of research studies has resulted in missed opportunities to reduce persistent cancer disparities. The conclusion offers recommendations for how to better document and use information on social factors in cancer research and care by (1) increasing education and awareness about the importance of inclusion of social factors in clinical research; (2) improving testing and documentation of social factors by incorporating them into journal guidelines and reporting stratified results; and (3) including social factors to refine extant tools that assess cancer risk and assign cancer care. Implementing the recommended changes would enable more effective design and implementation of interventions and work toward eliminating cancer disparities by accounting for the social and environmental contexts in which cancer patients live and are treated.

## Introduction

In 2015, a ground-breaking study highlighted how attention to two social factors, race/ethnicity [1, 2] and socioeconomic position (SEP), led to a key discovery in mortality trends in the United States (US) that had been masked for years [3]: All-cause mortality rates had been steadily declining in the US between 1999 and 2013; however when mortality rates were stratified by race and SEP, it became apparent that midlife mortality was actually increasing among one particular social group, Non-Hispanic Whites with low-education [3]. This example of contrasting findings by race/ethnicity, which in the US includes categories of Hispanic/Latinx or non-Hispanic Latinx^1^ White, Black/African-American, Native American, Asian and Pacific Islanders and others, and SEP demonstrates that attention to social factors in research points us to conclusions that might have been missed had those social factors not been explored. Breast cancer risk and survivorship research also offers several examples of disparities by race/ethnicity and other social factors that might otherwise have remained masked by the overall lower breast cancer incidence rates for most racial/ethnic minority groups compared to Non-Hispanic White women [4, 5]. Contrasting findings by race/ethnicity, SEP, and other social factors are present across the cancer continuum.

For example, recent findings suggest an increased risk of ER- breast cancer incidence among women born in states with Jim Crow laws (post-slavery laws in place in the US from the 1870s to 1964 that limited Black advancements and freedoms) [6]. Despite higher overall breast cancer mortality rates for Black women [4], both Black and Non-Hispanic White women with high education and who live in low SEP neighborhoods have similar mortality [7]. Early childhood abuse and neglect, which is more common among Black children than White [8], have been associated with elevated markers of inflammation among breast cancer survivors completing primary breast cancer treatment [9, 10]. These findings reinforce how recognizing social risk factors in cancer etiology research points us to inferences or novel hypotheses that might have been missed otherwise.

In this commentary, we describe social factors, and then use examples in breast cancer to demonstrate how the failure to appropriately incorporate social factors into the design, recruitment, and analysis of research studies has led to missed opportunities to reduce persistent cancer disparities. Greater attention to social factors across the breast cancer continuum is needed, given that differences in tumor biology and genetic variants have not completely explained the persistent Black/White breast cancer mortality disparity [11]. First, using an example of breast cancer risk assessment, we identify how excluding and then including the social factor of race [1, 2] has changed conclusions about eligibility for a breast cancer risk reduction strategy. Then, in two additional examples from breast cancer survivorship studies, we point to how the treatment and reporting of social factors have led to missed opportunities to address disparities. Finally, we offer recommendations for how to better document and address the cumulative effect of social factors and experiences that patients face.

### What are social factors?

Social factors include non-biological individual-level factors that influence health, such as race/ethnicity and SEP, as well as “upstream” community and societal-level factors [10, 12]. SEP is an aggregate latent construct that includes both resource-based (income, wealth, consumer credit) and prestige-based (education, social status) measures that represent both individual social position and access to material goods [13]. While race/ethnicity and SEP are the focus of this commentary as the most studied social factors in disparities research [14], we acknowledge that many other factors are significant across the cancer continuum: sexual identity and orientation [15–18], poverty and discrimination [6, 19], healthcare system distrust [20–22], health insurance quality [23], geography [19], nativity or immigration status [24–28], and housing [29–31], among others. Social factors of clinical relevance have been documented at the individual-level, interpersonal-level (e.g., patient and provider communication), and systems-level, extending to the societal-level and beyond [32, 33]. Further, some of these factors may change over the life course, with periods of exposure playing an important role for later cancer-related health risk [34]. Despite the body of evidence supporting the importance of social factors to cancer, incorporating social factors such as patient race/ethnicity and socioeconomic position into cancer research and clinical practice continues to be a challenge in both risk assessment and survivorship research [35]. For example, focusing on race/ethnicity has faced criticism because it is not a “modifiable factor,” while poverty and socioeconomic indicators face criticism because they are beyond the scope of health practitioners to treat. Nonetheless, race/ethnicity and SEP are associated with differential social, political, cultural, and economic experiences which can be modified and can influence cancer risk and care [10, 36].

### Opportunities to focus on social factors: example from breast cancer risk assessment

The history of the Breast Cancer Risk Assessment Tool, previously known as the Gail model [37], exemplifies the potential for information on social factors to reduce cancer disparities. The Gail model, which emphasizes non-genetic risk factors [38], is one of the foundational breast cancer risk assessment models and remains in use. It has been validated in many populations internationally [39], and played a substantial role in determining high-risk breast cancer populations to target for chemoprevention trials and additional screening in the United States [40]. The original Gail model was validated only in White women over the age of 35 [41, 42] and best estimated breast cancer risk for women who participated in regular mammographic screening [43]. Meanwhile, the model greatly underestimated breast cancer risk for Black women [44]: whose social grouping and experiences make them more likely than White women to develop breast cancer at younger ages; who have historically had low access to routine screening [45]; and who were under-represented in clinical trials preceding the development of the Gail model [46]. The original model’s underestimation of breast cancer risk among Black women precluded discussions that clinicians would have with Black women about eligibility assessments for tamoxifen chemoprevention trials [40].

In 2015, over 25 years after the initial Gail model was published, it was modified to explicitly account for Black race. The revised model more accurately estimated the expected number of breast cancer cases for Black women over age 30—narrowing the prediction for expected cases to be within 4% of the number of actual observed cases [46]. These revised models now estimate Black female eligibility for chemoprevention at nearly three times the rate of the original Gail model, increasing the estimated percentage of Black women eligible for chemoprevention trials to 17.1%, up from an earlier value of 5.7% [47]. Future risk assessment models may be in danger of repeating the Gail model’s history if they are not tested in other race and social risk groups and if they fail to include social factors that contribute to differential breast cancer risk assessments.

### Missed opportunities in analysis and reporting: example of breast cancer-related lymphedema

Overlooking differences by social factors also exacerbates disparities on the survivorship end of the breast cancer continuum. Descriptive analyses unadjusted for race suggest that Black breast cancer survivors when compared to White breast cancer survivors have higher incidence of breast cancer-related lymphedema, a persistent adverse effect of treatment that affects up to 35% of breast cancer survivors [48, 49]. This finding has been missed in regression models that evaluate risk factors for breast cancer-related lymphedema, such as obesity and hypertension, which are also more prevalent among Black women than White women [50–53]. Consequently, these differences by race and other social factors remain unaddressed in statistical models from which inferences for clinical practice are made. Until this issue is addressed, we fail to fully acknowledge that the obese Black cancer survivor simultaneously lives the experience of being Black, obese, and at higher breast cancer-related lymphedema risk, when in fact Black women (or any other women, for that matter) do not live their lives “adjusted” for race and obesity. In the words of Galea and Link: “What does it mean to enter a parallel universe wherein everything is the same except for one’s race?” [54, 55] The same rationale can be applied for social factors other than race.

While adjusting for social factors is appropriate if one is trying to isolate the contribution of a specific mechanism or exposure, this approach overlooks variations by social factors as a critical piece of information. Reporting analyses of results within social groupings can point to how social factors influence patient outcomes, which can lead to the development of effective interventions to help reduce existing disparities. Of course, that requires that data on social factors be collected in the first place, which is not always the case. A 2014 review of over 20 years of National Cancer Institute (NCI) clinical trials found that as few as 1.5–58% of studies reported results on race/ethnicity and that only 20% of randomized controlled studies reported results stratified by race/ethnicity [56]. More recently, an as-yet unpublished analysis by this paper’s authors examined the 57 breast cancer observational and randomized controlled trials that were published in a major cancer clinical journal in 2016. Fewer than half, 44% (*n* = 26), reported descriptive information on race/ethnicity and fewer than 25% (*n* = 13) reported the social or economic composition of their study samples (unpublished data compiled by study authors solely for the purpose of this commentary). Excluding those in which the primary focus was disparities, fewer than 5% reported findings stratified by race or other socioeconomic factors. If health disparities are to be addressed, they must first be identified. Collecting information on social factors, and then exploring stratified results by those social factors, can help identify disparities and directly inform strategies for their elimination.

Stratification may lead to different conclusions than those that would be drawn looking at trends across the full sample. Simpson’s paradox occurs when the trend seen in the aggregated data does not hold in the stratified homogenous groups [57]. For example, although Asians in general have historically had lower breast cancer mortality compared to Non-Hispanic Whites [4], stratifying by country of origin shows that Filipina women have worse 5-year survival rates for late-stage disease than Whites, while Japanese women have more favorable rates [28]. Having this information might suggest that Asian subpopulations need special attention, which might have been missed without examining within race strata.

### Missed opportunities in intervention due to lack of attention to social factors: example of physical activity for breast cancer survivors

Social factors also need to be addressed when assigning follow-up care to ensure that it is appropriate, reasonable, and accessible for a patient. Physical activity interventions are increasingly recommended as part of effective cancer survivorship care plans [58] to reduce cancer-related fatigue, and to promote cardiopulmonary function and weight control [59–62] among women with a history of cancer. Physical activity interventions have been less successful among Black women when compared with other racial/ethnic groups, largely due to lack of attention to the social factors that frame their health behaviors, including where they live and the demands on their time and resources [63]. For example, a physician’s recommendation of increased physical activity after cancer treatment will fail unless the patient’s barriers to physical activity are addressed, such as ensuring safe places to exercise or access to a gym facility [64]. Failure to address these factors likely contributes to disparities in quality of life, and thus risk factors for breast cancer recurrence persist for Black women, and may contribute to their shorter survival times after cancer treatment [11].

### Social factors can be meaningfully incorporated into cancer research and practice

Although the examples in this commentary were from breast cancer, the implications are not specific to breast cancer, and the recommendations can be applied to cancer research more broadly. In light of these examples, we make three recommendations for how cancer research and practice can better incorporate social factors into research and practice: (1) increase education and awareness about the importance of inclusion of social factors in clinical research; (2) improve testing and documentation of social factors by incorporating them into journal guidelines and reporting stratified results; and (3) include social factors to refine extant tools that assess cancer risk and assign cancer care.

### Recommendation: increase education and awareness among researchers and health providers of the importance of inclusion of social factors

Attention to social factors in clinical care and at the community level begins with clinical research that meaningfully accounts for a diverse sample of patients. Higher recruitment and accrual of racial/ethnic minorities from low SEP backgrounds who are underrepresented in clinical trials [35, 65, 66] will allow researchers to focus on how race and modifiable social factors may be primary exposures that drive disease patterns. This work may necessitate additional resources for demographic data collection with real-time tracking of accruals [67] and targeted recruitment for the larger sample sizes this will require [66], as well as commitment to diversity from individuals, teams, and institutions [67]. Having diverse samples that allow for documentation of race-based disparities is critical to continue to emphasize, especially given recent calls by members of the U.S. Congress to exclude data collection on racial disparities^2^.

When there is scientific evidence or a theory-driven hypothesis to do so, investigators should consider expanding their research questions to examine social factors explicitly and consider including investigators with expertise in social epidemiology, community-based participatory research, and health disparities. It is especially important to use quantitative and qualitative approaches and statistical tools [68–71] that are designed to explore differences by race, or other social factors (e.g., ethnicity, immigrant status, SEP) [72, 73]. For example, analyses might include hierarchical linear modeling and/or geospatial analyses to disentangle the role of environmental social factors (e.g., neighborhood access to health services) that influence health outcomes for individual cancer survivors embedded within these contexts. Propensity scores could be used in regression analysis in situations in which accounting for a high number of clinical and social factors is required. The Peters-Belson method (called the Blinder–Oaxaca approach in economics) is most commonly used to determine wage discrimination but has many applications to the study of health disparities [74–76]. The Peters-Belson method estimates the proportion of an overall disparity that is not explained by the covariates in the regression. This method first fits a regression model with individual-level covariates to the majority/advantaged group and then uses the fitted model to estimate the expected values for minority-group members had they been members of the majority group [69, 71]. Using qualitative interview data or focus group data can often reveal why research results are what they are, beyond what the effect size of an odds ratio or hazard ratio can ever reveal.

Efforts are likewise needed to expand the collection of data on social factors beyond race to other factors of significance across the cancer continuum, such as sexual identity and orientation [15–18], poverty and discrimination [6, 19], healthcare system distrust [20–22], and geography [19]. Some health systems have already started incorporating data on social factors into electronic health records [77], which could have the dual purpose of expanding research opportunities to link social factors to individual health outcomes and biomarkers, and informing clinical practice. It might later allow providers to develop treatment plans that recognize and address patients that may be facing adverse social conditions that undermine treatment success (e.g., food insecurity, poor geographic accessibility).

### Recommendation: improve testing and documentation of social factors by incorporating them into journal guidelines and reporting stratified results

Both measurement and reporting of social factors remain important because the sociocultural classification of individuals in our society has resulted in differential health practices and outcomes. Exposure to depressed and segregated economic and residential environments, poor access to health care, poor access to quality education, and greater exposure to psychosocial stressors, can all impact cancer risk, treatment receipt and whether there is partial or complete pathologic response [36, 78]. Despite calls for increased accountability in reporting social factors [79], suboptimal measurement and reporting of social factors persist in published studies in cancer [79]. This suboptimal reporting and measurement may mask social differences or inequalities that, if reported, could be addressed to optimize patient care and survivorship. When reporting data on social factors, reviewing results to examine the distribution of pre-existing social factors is a simple first step in understanding and addressing social context.

When possible, findings should stratify these data by race, SEP, country of origin, sexual orientation, health insurance, or other social factors that theory or previous research suggest are important to the exposures or outcomes being studied. This examination is essential for the identification of those social factors that are the most salient. Current journal publication reporting checklists, including the CONSORT checklist for clinical trials that is used in over 50% of core medical journals (http://www.consort-statement.org/about-consort/endorsers), and STROBE and RECORD checklists for observational studies [66, 80–82], call for reporting of demographic data. Yet, none is prescriptive about reporting race/ethnicity or other social factors specifically, or about conducting sub-analysis by groups that are historically underrepresented in medical research. Assessing interactions among social factors and conducting stratified analysis when interactions between variables that are found to be significant are important for ensuring that study conclusions hold for the entire sample. Specifying regression models for specific groups, and comparing parameter estimates for the same variables in each of the models are recommended [83]. Conducting stratified analysis in this way allows for inclusion of different variables in the models for different groups based on what is most relevant, in contrast to interaction models which assume the same predictors and confounders across all groups. More specific reporting on how social factors influence patient outcomes can help translate research to clinical care, by guiding how study results might be translated into practice.

### Recommendation: use social factors to refine tools that assess cancer risk and assign cancer care

As the body of data on social factors grows, and reporting of these factors increase, this information may be used to refine tools for cancer screening, care, and survivorship. Similar to the way in which the Gail model was adapted to include race information, treatment risk profiles might be expanded to include other elements of social context. In survivorship care, the recent Commission on Cancer and National Accreditation Program for Breast Centers’ accreditation requirement of Survivorship Care Plans offers yet another opportunity to incorporate the role of social factors. The success of the care plans will depend on the ability of health providers and health systems to ensure that each patient’s care plan goal is achievable, and will confront the reality of designing a care plan that allows patients to successfully navigate their social lives and experiences to achieve healthy outcomes.

## Conclusion

Incorporating social factors such as patient race/ethnicity and SEP into cancer research and clinical practice continues to be a challenge in both cancer risk assessment and survivorship research [35]. For example, trying to resolve disparities by “race/ethnicity” has fallen under criticism because race is not a “modifiable factor” and the social experiences associated with race, such as poverty and SEP, are often beyond the treatment scope of health practitioners. Nonetheless, race/ethnicity and SEP are associated with differential social, political, cultural, and economic experiences that can be modified and can influence cancer risk and care across the cancer control continuum [10, 36]. By acknowledging a range of social risk factors, the changes that we have recommended would enable more effective interventions to be conducted. Furthermore, they would work toward truly eliminating disparities by accounting for the social and environmental contexts in which cancer patients live and are treated.

## Footnotes

1 Latinx is a gender-neutral term for Latinos.

2 In January 2017, bills were introduced in the US Senate and House of Representatives to restrict the use of Federal funds “to design, build, maintain, utilize, or provide access to a Federal database of geospatial information on community racial disparities or disparities in access to affordable housing.” [http://www.congress.gov/bill/115th-congress/house-bill/482; http://www.congress.gov/bill/115th-congress/senate-bill/103]. Bills with nearly identical language had been introduced into Congress in 2015 as well.
